# Recipe Components and Parents’ Infant and Young Child Feeding Concerns: A Mixed-Methods Study of Recipe Posts Shared in Thai Facebook Groups for Parents

**DOI:** 10.3390/nu13041186

**Published:** 2021-04-03

**Authors:** Abhirat Supthanasup, Cathy Banwell, Matthew Kelly, Vasoontara Sbirakos Yiengprugsawan

**Affiliations:** 1 Research School of Population Health, Australian National University, Acton, Canberra 2601, Australia; cathy.banwell@anu.edu.au (C.B.); matthew.kelly@anu.edu.au (M.K.); v.yiengpr@unsw.edu.au (V.S.Y.); 2 School of Human Ecology, Sukhothai Thammathirat Open University, Nonthaburi 11120, Thailand; 3 Australian Research Council Centre of Excellence in Population Ageing Research, University of New South Wales, Kensington, Sydney 2033, Australia

**Keywords:** social media, Facebook, infants and young child feeding, food choice, mixed-methods, Thai

## Abstract

Social media is increasingly becoming a significant source of information for parents, including about feeding young children. However, little attention has been given to the characteristics of recipes for infants and young children and how they interact with parental perceptions regarding food decisions shared by users on social media. Building on findings related to shared recipe components and parental food choices, between December 2019 and July 2020, this study retrospectively collected 80 shared recipes each from five Thai Facebook groups. This extraction created 379 shared recipes with 1751 peers’ commentaries on the shared recipes’ posts. The shared recipes were classified and components quantified across child age groups, then the textual contents around the reasons behind the food choices were described qualitatively. The results showed that there were differences in meal types, food ingredients, and seasoning used across child age groups. Further analysis found that food allergy awareness was one driving concern behind parental perceptions on food choices in children’s diets. These concerns resulted in delays in the introduction of animal-source foods. Moreover, peers’ commentaries on shared recipes offered a venue for exchanging experiences with food products. Because of the potential influence on parental beliefs and perceptions, further studies are required to understand the impact of existing online communities on actual feeding practices.

## 1. Introduction

Although infants’ and young children’s diets play a significant role in promoting good health, providing appropriate feeding can be fraught with many challenges for parents and caregivers. The transition from exclusive breastfeeding to a family diet is a period characterized by rapid growth and development, which is highly vulnerable to nutrient imbalance. This period plays an essential role in preventing malnutrition (both under- and overnutrition) and its long-term adverse consequences and promoting healthier growth and development [1]. However, nowadays, foods for young children usually fall short of providing diversified and nutrient-dense foods [2]. Global estimates show that less than one in three children aged 6–23 months are eating at least 5 of 8 food groups [3], whereas in East Asia and the Pacific, around three in five children fail to reach this minimum number of food groups [3].

Moreover, a rapid nutrition transition, especially in low and middle-income countries, has contributed to poor-quality diets of high-calorie, low-nutrient, processed foods. Diets that are low in iron-rich foods, fruits, and vegetables are common problems among infants and toddlers [4]. Time constraints and convenience are found to influence parents’ food choices toward obesogenic dietary intakes, such as fast food, junk food, convenient food, and sugar-sweetened beverages [5]. The Pregnancy and Birth to 24 Months (P/B24) project found an association between type of complementary foods and micronutrient status and the risk of obesity and allergy in childhood [6]. As diet quality is one of the essential drivers of malnutrition [7], poor diversity and low-quality diets increase the risk of multiple forms of malnutrition. Given these significant changes in dietary pattern and development, food choice is crucial in avoiding imbalances in nutrient intake, such as insufficient iron and excessive amounts of other nutrients such as sodium. Parental food choice for their children not only impacts on child nutrition and health status but also influences food acceptance and future eating habits [8]. Therefore, understanding modern social influences on parental feeding choices and perceptions about what, how, and when around young child feeding is needed.

There are many factors that drive parental decision making around feeding choices for their infants and children. One emerging and understudied factor that is important when trying to improve understanding of these decisions is the increasing influence of social networking sites, which are now helping facilitate the emergence of new landscapes of health and society. In Thailand, in 2020, around three-fourths of the total population were active social media users [9]. The rise in popularity of social networking sites has led to an increased interest in them as a health information source, giving them significant potential to influence parental perceptions and food decisions about child feeding [10].

There is a growing body of literature regarding social media and parenting, including child feeding practices. Some studies focused primarily on applying interventions using social media as communication tools for parents. For example, studies on healthy lifestyle promotion on social media found no evidence that parents’ exposure to these online interventions improved their children’s fruits and vegetable consumption and body mass index [11,12]. Other studies have investigated the child feeding information shared by parents on social media [13,14,15]. One recent study found that sharing food recipes for children is a common parenting topic on social media, especially in Facebook groups [13]. Work investigating the so-called “mom food blogs”, covering child feeding practices and shared recipes, is still preliminary [14], although we do know that parents are heavily engaged on social media [16,17] and value peer support from online communities [18]. A previous study found that parents accessed social media primarily for information seeking and advice [19]. Because social media sites enable parents to share views and information about the type and amount of food children should consume [10], they offer valuable yet relatively underutilized access into shared views on feeding children, which can be explored through further research.

The current study aimed to describe the characteristics of parental food choices and perceptions of child feeding through assessing user-generated content around shared recipes for infants and young children disseminated by Facebook groups. Specifically, the first objective was to describe the recipe type, meal combination, and vegetable variety in shared recipes across different age groups of infants and young children. The second objective was to investigate the ingredients used in shared recipes. The third objective was to explore peer-to-peer discourse in response to shared food recipes that may shape parental child feeding beliefs and perceptions.

## 2. Materials and Methods

### 2.1. Research Procedure

To identify the eligible Facebook groups that address children’s diets, four search phrases, “healthy diets for children” (อาหารสุขภาพสำหรับเด็ก), “food for children” (อาหารสำหรับเด็ก), “children’s menu” (เมนูสำหรับเด็ก), and “children’s meal ideas for new mother” (ไอเดียอาหารเด็กสำหรับแม่), were used for searching in Facebook on 9 and 10 December, 2019. These four search phrases were formulated based on the keywords of the reported names of social networking sites used as a source of child feeding information for Thai parents from an unpublished previous survey. Each single keyword was then incorporated into the multiple keyword strings and initially tested with Facebook’s built-in group search. These final four search phrases were selected in order to identify a comprehensive and large number of relevant groups. This search generated 39 relevant Facebook groups. Membership ranged from 1 to 250,000 community members. The groups with more than 1000 members were reviewed. The inclusion criteria were (1) the group is administered by a parent of Thai children (not an organization, company, or healthcare professional), (2) written in Thai language, and (3) focuses on a peer discussion of diets for children. Introductory messages and information sheets were sent to the administrators to seek approval to collect data from the group postings. Of the 20 administrators of eligible groups who were contacted, seven responded, and five approved data collection.

To create the dataset, between December 2019 and July 2020, the most recent 40 shared recipes for children aged under 1 year old and 40 shared recipes for children aged 1 year and older and their comments were extracted retrospectively from the five Facebook groups. The inclusion criteria of eligible recipes were (1) indicate the target age-group for the recipe on the initial post description by poster, (2) must state the list of ingredients and cooking methods, and (3) has to be a main meal, not a snack (defined as food eaten between meals, usually self-fed, convenient, and easy to prepare, such as finger food, piece of fruit, or bread) or dessert. Identities of group members or group names and any identifying information were not recorded. Project information was posted on the Facebook groups so that group member could request that their posts not be included if they had concerns. Group administrators, as a representative of the community, could also report any concerns or check the dataset to ensure that confidentiality was protected during the data collection. This resulted in a database of 379 shared recipes and 1751 comments. The shared recipes were classified and the components quantified across child age groups. Textual content in the initial posts’ description and comments were then analyzed qualitatively.

### 2.2. Quantitative Data Analysis

Food ingredients were classified by the first author, who is a trained nutritionist, based on the sample food list in the World Health Organization guide for assessing infant and young child feeding practices [20]. This provided a comprehensive food list as our dataset. Only a few Thai native vegetables were classified and added to the food list by the first author based on his experience as a trained nutritionist in Thailand. Food categories, such as grains, dark green leafy vegetables, dark yellow or orange fruits, and organ meats, were then combined into eight food groups according to the WHO guidelines on dietary diversity [21], which are (1) grains, roots, and tubers; (2) legumes and nuts; (3) dairy products; (4) flesh foods (meat, fish, poultry, and organ meats); (5) eggs; (6) vitamin A-rich fruits and vegetables; (7) other fruits and vegetables, and (8) breastmilk. An additional two food groups of (9) oils, fats, and butter, and (10) condiments were also created. A vegetable variety score (VegVS) was calculated using the number of vegetable varieties within shared recipes to examine the food variety exposure [22,23]. As all shared recipes only included ingredients but not quantity, no limitation to the quantity of food was applied for the score calculation. Consequently, nutritional values were not able to be calculated in this study.

Chi-square tests and ANOVA were performed to assess whether the type of food ingredients and VegVS were significantly different based on five children’s age groups, 6–7 months, 8–9 months, 10–11 months, 12–23 months, and 24 months and over. Statistical analyses were conducted with IBM SPSS Statistics (version 25.0 for Windows). Significance was accepted at *p* < 0.05.

### 2.3. Qualitative Data Analysis

Inductive thematic analysis [24] was used to explore the peer-to-peer discourse in the initial post descriptions and comments sections under the shared recipes, concentrating on child feeding beliefs, practices, and concerns. The first step in the analysis was to become familiar with the data by reading and re-reading the dataset. Initial codes were then developed inductively without trying to fit them into a pre-existing coding frame. Similar codes were then combined into themes. The first author was responsible for developing the initial codes. These, with selected excerpts and emergent themes, were then discussed with other authors until the consensus was reached to ensure the credibility of the study [25]. Upon completion, the themes were reported with illustrative quotations.

## 3. Results

### 3.1. Quantitative Results: Characteristics of Shared Recipes

Table 1 presents the characteristics of the 379 shared recipes. Overall, ninety percent of shared recipes contained animal-source food, which consisted of at least one ingredient from flesh foods or egg or dairy. Recipes containing non-animal source foods were more commonly aimed for younger children, aged 6–7 months old. The proportion of recipes containing animal-source food increased with children’s age (*p* < 0.001). Among the combinations of shared recipes, the most frequent contained animal-source foods (flesh foods/egg/dairy) + grain/legume + vegetable/fruit (59.6%, *n* = 226). The proportion of recipes without vegetable/ fruit increased with children’s age. Regarding the vegetable variety, the overall shared recipes had a vegetable variety score (VegVS) at 2.0, meaning they contained two types of vegetables. Across the age range, the VegVS of shared recipes for children aged 10–11 months old was slightly greater than that of recipes of older- and younger-aged children. Moreover, the interquartile range (IQR) of VegVS was larger in shared recipes for the youngest children, which reflect that the vegetable variety seemed to vary more between each recipe in this group. Whereas, the IQR of VegVS was smallest in shared recipes for the oldest group, with median VegVS of 1.0. This means that, generally, shared recipes for children aged 24 months and older used only one type of vegetable.

### 3.2. Quantitative Results: Trends of Food Groups and Ingredients Used in Shared Recipes

#### 3.2.1. Trends of Food Groups Used in Shared Recipes across Children’s Age Groups

**Figure 1:**
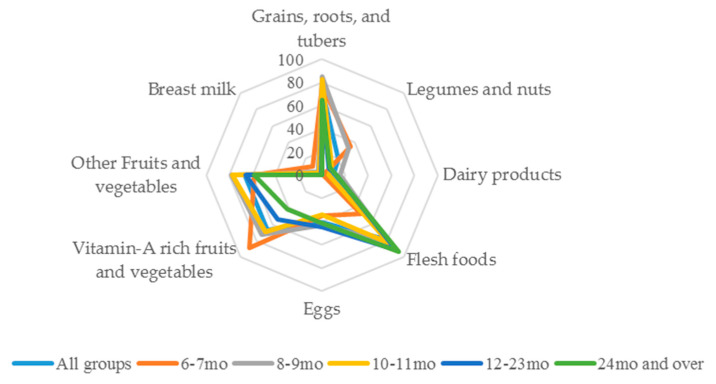
Trends of the percentages of food groups used in shared recipes across children’s age.

The eight food groups, according to the WHO guidelines, used in shared recipes were compared across children’s age groups. Among flesh foods, eggs, and dairy as animal- source foods, the comparison indicated that there were trends in the increasing use of flesh foods (meat, fish, poultry, and organ meats) with increasing children’s age. Overall, flesh meats were used in around 80% of shared recipes. However, there was a smaller percentage using flesh meats in shared recipes for children aged 6–7 months compared with 8–9 months, 10–11 months, 12–23 months, and 24 months and over, which were 47.3%, 82.4%, 82.9%, 90.5%, and 93.5%, respectively. Dairy products, such as cheese and cow’s milk, were uncommonly used among shared recipes for younger-aged children, 6–7 months old. In turn, there was an increased use of dairy products in shared recipes by eight months of age, whereas there was a small difference in the use of eggs in shared recipes across age groups. The use of legumes and nuts, grains, roots, and tubers, and both groups of fruits and vegetables in recipes decreased with children’s age. For example, the use of vitamin A-rich fruits and vegetables was common in shared recipes for children aged 6–7 months. The percentage of use then significantly decreased in the shared recipes for older children. Breast milk was commonly used in recipes at the early stage of complementary feeding, with a decline in use when children get older (Figure 1).

#### 3.2.2. Trends of Ingredients Used in Shared Recipes across Children’s Age Groups

**Figure 2:**
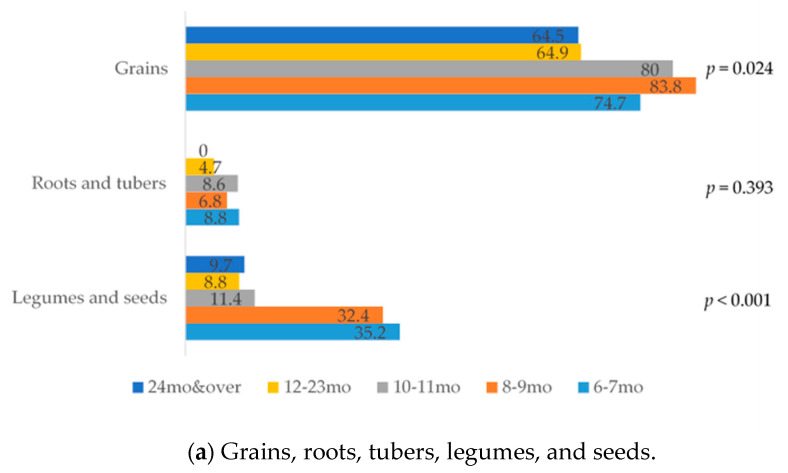

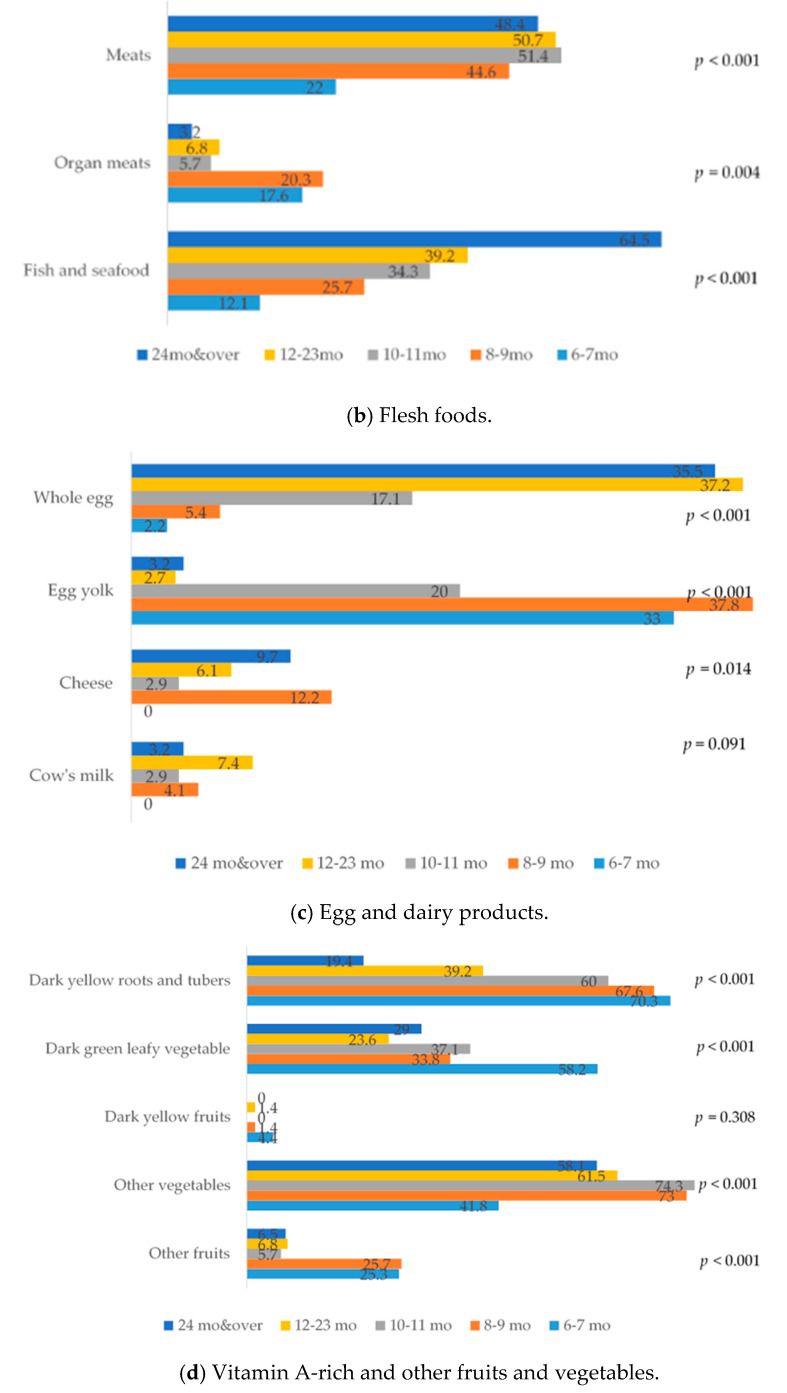
Percentage of ingredients used in shared recipes across children’s age groups.

**Figure 3:**
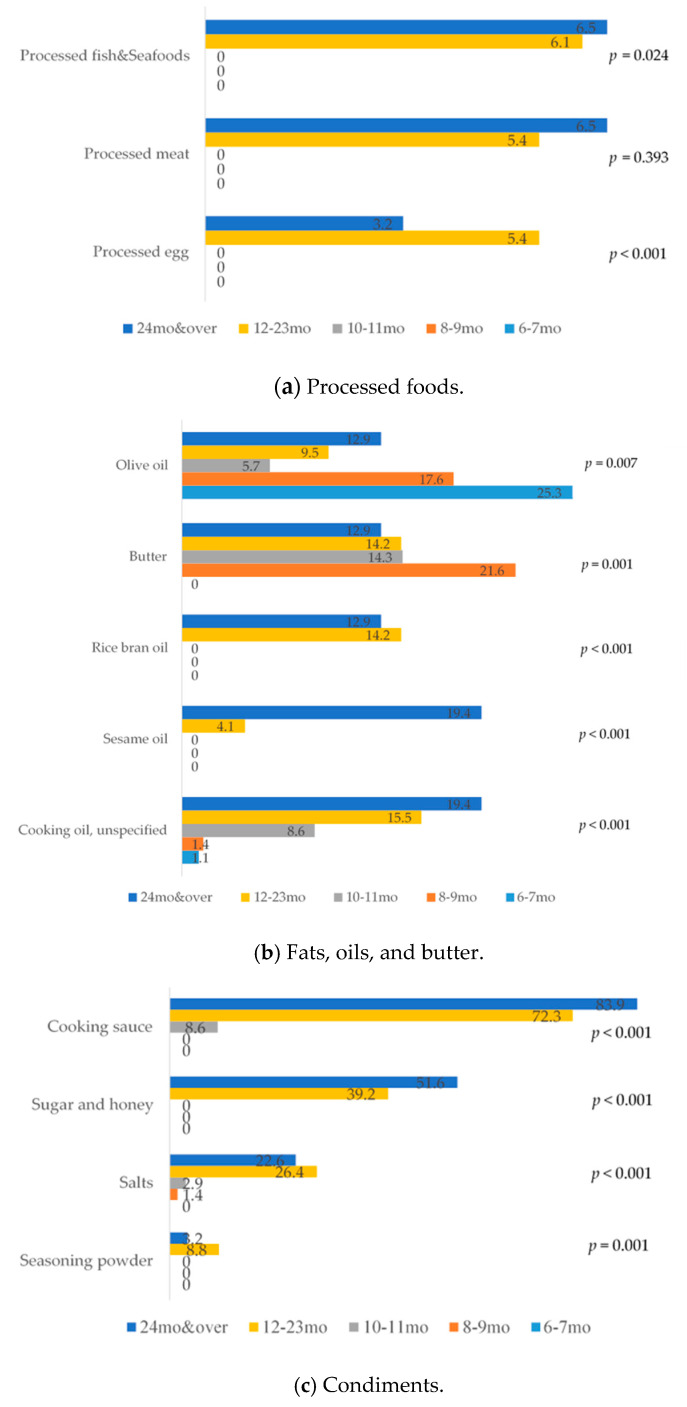
Percentage of ingredients used in shared recipes across children’s age groups (continue).

Figure 2 and Figure 3 display trends of ingredients used in shared recipes for infants and young children across several age groups.

(i) Grains, roots, and tubers. In this group, grains were primarily used in shared recipes, whereas very few used roots and tubers (Figure 2a). Rice was predominantly used in shared recipes in all age groups; around half of the shared recipes for children under one year old used rice. The use of rice declined in shared recipes for children as they get older; with increasing use of wheat-based bread and noodles. Among nontraditional staple foods, quinoa, oat, and potato were used in recipes for younger-aged children and used markedly less for children aged 10–11 months and older.
(ii) Legumes and nuts. Legumes and seeds were used in nearly one-third of all shared recipes for children aged 6–9 months. However, the percentage use decreased to lower than 12 percent among shared recipes for older age groups (Figure 2a). Sesame seeds, flaxseeds, and sugar snap peas were the top three ingredients for this group. Whereas seeds and legumes were commonly used in complementary food, nuts were never mentioned in any shared recipes.
(iii) Flesh foods. Meats, especially pork and chicken, were most used in this group; however, there was a relatively small number of meats used in shared recipes for younger children, aged 6–7 months. Whereas organ meats, especially liver, were highly used in shared recipes for younger children, aged 6–9 months, after this age range, the use of organ meats declined. Fish and seafood were highly used in recipes for older children, of which prawn, salmon, and seabass were the top three. The percentage of shared recipes using fish and seafood, especially prawns, increased sharply in recipes for children aged 2 years old and over (Figure 2b). Processed meats and fish, such as ham, sausage, and fish balls, began to appear in shared recipes for children aged one year and older (Figure 3a).
(iv) Eggs. The use of egg yolk was highest among shared recipes for younger children, aged 6–11 months old. The whole egg was uncommonly used for younger-aged children, whereas there was an increase in use for older age groups (Figure 2c). There were a few recipes which used only egg white.
(v) Dairy products. Dairy products were uncommon among shared recipes for younger-aged children. However, by eight months of age, cheese and cow’s milk were increasingly used (Figure 2c). A few recipes used yogurt.
(vi) Vitamin A-rich fruits and vegetables. In this group, dark yellow- or orange-fleshed roots and tubers and dark-green leafy vegetables were commonly used in shared recipes for young children when compared with dark yellow and orange fruits (Figure 2d). Carrot was the most commonly used from this category of vitamin A-rich fruits and vegetables, followed by pumpkin and broccoli.
(vii) Other fruits and vegetables. Other vegetables were commonly used in shared recipes. The use of other fruits and vegetables declined by children’s age (Figure 2d). Onion, tomato, and baby corn were the top three in this group.
(viii) Oil, fats, and butters. The most common oil used in shared recipes was olive oil. Olive oil was significantly more commonly used in complementary food recipes for younger children aged 6–9 months with a remarkable decrease in use in recipes for older age group’s recipes, whereas rice bran and other oils were used in recipes for older age groups. Moreover, butter was introduced in recipes for child aged from 8 months old (Figure 3b).
(ix) Condiments. Condiments were not mentioned in any shared recipes for younger children aged 6–7 months (Figure 3c). The use of condiments was prevalent in recipes for children aged 1 year and older. Soy sauce, salts, and sugar were the most commonly used food seasonings.

### 3.3. Qualitative Results: Peer-Exchanged Discourse on Shared Recipes around Child Feeding Beliefs, Practices, and Concerns

From the study, there were four emerging themes on child feeding beliefs, practices, and concerns around shared recipes for infants and young children. (1) Introducing common allergenic foods to children with age-appropriate awareness. (2) Making a plan to introduce new food. (3) Seasoning is only introduced once your child is 1 year old. (4) Peer recommendations build confidence in specific food products. Details of these are given below:

#### 3.3.1. Introducing Common Allergenic Foods to Children with an Age-Appropriate Awareness

Age-appropriate food awareness was one of the topics of most interest among group members. Food allergy awareness consistently elicited parental views on food decisions across different age groups of infants and young children. Specifically, shellfish, egg white in whole egg, and dairy products were often mentioned when responding to food allergy concerns. Group members expressed the opinion that it is best to delay shellfish introduction until children are aged 2 years and older. They were highly concerned about the prevalence of shellfish allergy and the age appropriateness of the recipes:

“High prevalence of shellfish allergy. So shrimp and crab should start from 2 years old.”

“Please indicate clearly in your shared recipes that this shrimp recipe is suitable for children 2 years old and older.”

As with shellfish, there were concerns that the child should be of appropriate age at the first introduction of egg white. To avoid the risk of egg white allergy, egg yolk was often mentioned as a suitable choice for preparing meals for children aged under 8 or 12 months old:

“I’m sharing my stir-fry quinoa with zucchini and egg. Notably that use only egg yolk if your child under 8 months old.”

“If you are preparing meal for your child under 1 year old, you should use only egg yolk and do not season.”

Many dairy product-containing recipes indicated clearly that these meals were appropriate for children aged 8 months and older:

“Mashed chicken’s liver with apple and cheese (suitable for children aged 8 months and over without milk allergy)”

“Sweet potato with egg, watercress and cheese (This recipe contains cheese and butter which suitable for children aged 8 months and over)”

#### 3.3.2. Making a Plan to Start New Food

Apart from the potentially allergenic foods discussed above, it was clear that many group members were concerned whether their child might develop a food allergy and intolerance when introduced to new foods. These concerns contribute to the practice of making a plan to start new food for the first time. There was no clear regimen disseminated through the groups; however, there was some advice on the appropriate way to start feeding new food for the first time. Group members encouraged each other to start first on solid foods with rice mixed with breast milk followed by the introduction of other new foods for around 3–5 consecutive days to see whether an allergenic reaction developed:

“Start the first solid food with rice mixed with breast milk, then add leafy vegetable in the next meal.”

Parents employed scientific language:

“After passing the test of rice with breast milk, you should test the leafy vegetable before root vegetable (such as pumpkin, carrot). You should test each food 3–5 days before introducing the new food.”

However, not all members were concerned about food allergy and shared their own experiences with a mixture of all food groups. Some parents shared their experiences of the benefits of offering a mixed dish so that their child did not suffer from micronutrient deficiencies:

“I give my 6-month-old child a mixed dish with all food groups. If there is no food allergy history, it’s not necessary to test every single food.”

“I have been giving my child a mixed dish with five food groups from 6 months old as a doctoral suggestion. I have no family history about food allergy. And my child has not suffered from anemia.”

#### 3.3.3. Start Seasoning Your Child’s Food from 1 Year Old

Seasoning food prepared for young children was one of the high concerns among community members. Children’s age plays an important role in determining parental perceptions on seasoning home-cooked food. It was clear that community members avoided adding sugar, salt, and other seasoning in home-cooked food for children age under 1 year old for health benefits:

“If your child is under one year old, I recommended you not seasoning your home-cooked food for your children. Giving unseasoned food to your young child will help to ease the workload of his/her kidney function.”

“Delaying food seasoning start time will benefit your child health. Food seasoning should start from 1 year and older.”

#### 3.3.4. Peer Recommendations on Food Products Build Confidence

Sharing and recommending food products was an integral part of peers’ discourse around sharing food recipes for infants and young children. Community members typically asked about, or questioned, unfamiliar food ingredients used in shared recipes. Specifically, shared recipes containing cheese, butter, or olive oil led to other members’ attention to food products. For example, community members always asked for further information on the cheese, butter, or olive oil brand:

“What cheese product did you use? Can you share a picture of the package?”

“Can you let me know the product name of butter you used in this recipe?”

To respond to the requests, a product name, sometimes with a picture of the package, was posted under the discussion thread. Cheese or butter selection was based on the ingredient list with high in milk, low in salt, and without food additive:

“I used this cheese product. Please see the product’s package below.”

“I used this cheese product [name of the product]. Basically, read the label and choose the product made from at least 98% milk with salt lower than 1% and no ingredient that has E-number, which is a code indicating food additives.”

“You can use any brand of olive oil, but it should be an extra virgin.”

Community members had conversations around unfamiliar food products, processed meats, and food seasoning. The relaxation of the constraints on added sugar, salts, and other food seasoning after the child reached a year led parents to focus on processed meats and food seasoning in shared recipes:

“Can I know the brand of crab stick you used in this recipe?”

“Which brand of soy sauce do you recommend? Where can I get it?”

“Could you please share the picture of alternative seasoning powder that you used in this recipe?”

The community discourse continued to focus on reduced sugar and salts and additive-free products even in processed meats and food seasoning:

“I used this sausage in my recipe because of it is nitrates and nitrites-free. But it still has MSG.”

“I used this low-sodium soy sauce [product name] in my recipe.”

## 4. Discussion

To our knowledge, this mixed-methods study is the first to characterize the food items used in online shared recipes across different age groups of young children, and it offers a deeper understanding of parental perceptions of these foods’ characteristics. Our results suggest that home-cooked foods are advocated among online members as all of the shared recipes were self-prepared, although they included processed foods such as sausages and bread. The composition of home-cooked foods is dependent on parental perceptions around food choice, which are influenced by both individual and external influences [26]. Online shared food recipes and peers’ discourse about them are part of the external social environment that shapes parental perceptions about the perceived appropriateness of home-cooked foods for infants and young children. The socialization process of modeling, reinforcement, and social interaction may influence behavior and intention [27]. Online communities, which enable users to contribute and observe user-generated content easily, are likely to facilitate social learning through the socialization process mechanism [10]. The online user-generated content, such as shared recipes and discourse about the recipes, may influence parental food decision making by providing information and establishing social norms to guide what is perceived to be appropriate food consumption practices [10]. Our study found that the threads around shared recipes could be viewed as a source of information about food products and brands. Whereas the flood of commonly observable food choices could establish a descriptive norm within an online group, our findings point toward a descriptive norm around age-appropriate food choice for infants and young children embedded within online-peer support groups. This perception of age-appropriate food choice is likely to influence other members in making a decision on home-cooked food for their children.

Allergy awareness was one of the predominant factors guiding age-appropriate food choice within shared recipes for infants and young children. Our analysis highlights that some common allergenic foods were significantly more likely to be introduced in recipes for older children. This finding is then confirmed by our thematic analysis, which underlined that the allergic potential of these foods was one of the reasons for the use of different foods in shared recipes across child ages. This supports a previous finding that parents avoided potential allergenic foods at the early phase of complementary feeding as a result of allergy awareness [28]. An analysis of online discussion forums found that parents were concerned about allergies; however, they were confused about the difference between allergy and intolerance [29].

Diet plays an important role not only as a source of energy and nutrients but also as an environmental factor contributing to food allergy. Over the years, the knowledge around the timing of allergenic food introduction to infants has evolved. The traditional recommendation has focused on delaying introduction of the potential food allergen due to structural and functional immaturity of children’s guts [30]. However, given the increasing prevalence of food allergies in the last decades, attention has shifted towards the early introduction of potentially allergenic foods to children during a critical immune developmental period to build tolerance [30]. A previous review has found that while delaying allergenic foods beyond 4 to 8 months of age was not associated with decreased risk for allergies in infants, the delay in introduction of these foods beyond nine months might increase the risk of allergy development [31]. The dual-allergen exposure hypothesis serves as a theory explaining that early ingestion would establish tolerance while delayed ingestion might increase the risk of food allergy [32]. Even so, our study revealed that the parental concerns about food allergy were the common driver of delaying some common allergenic foods beyond 8 months of age. However, despite the potentially modifiable cause of food allergies, and growing evidence of benefits in an early introduction to allergenic food, there is no clear evidence on the specific timing of food introduction for preventing allergic diseases in both high- and normal-risk infants [30].

Our study found that egg white in whole egg and shellfish were common concerns around allergenic foods among online members. Egg, shellfish, and cow’s milk allergies are common in many countries [33]. Interestingly, while the whole egg was predominately used in shared recipes from 10 months of children’s age, egg yolk was commonly used in shared recipes for younger-aged children. The reason shared recipes delayed whole egg introduction was a concern about egg white allergy. However, there is increasing evidence found that late introduction of egg, around ten months of age, is associated with an increased risk of egg allergy or sensitization [34,35]. A systematic review found that egg exposure from age 4 months reduced the risk of egg allergy and sensitization [36].

Our study also found that shellfish was commonly used in shared recipes for children aged two years old and over. The qualitative results revealed that the reason behind this trend is the parental concerns about shellfish allergy. What lies behind this serious concern may be the evidence that shellfish was one of the most common food allergens in preschool children in Thailand [37]. The immature immune system could help explain why shellfish should be avoided until after 2–4 years of age in high-risk children, however there is no evidence to support this recommendation [38]. Our discourse analysis also found the reason behind the delayed introduction of dairy products is concerns around cow milk allergy. Furthermore, nuts are not used in any shared recipes across all ages. It may be assumed that online members are also concerned about nut allergies as the most common allergen in children globally [39]. Surprisingly, although sesame was classified as an allergenic food along with egg white, shellfish, and milk [40], our study found that sesame was used in many shared recipes at the early stage of complementary feeding. The awareness around the type of food allergens among parents may differ by region, as sesame is one of the most common allergens in the Middle East countries [41].

Food allergy awareness is not limited to common allergenic foods but also occurs in relation to other foods. Allergy concern seems to be one of the factors in delaying the introduction of animal-source foods. Our recipe characteristic analysis found that 33% of recipes for children aged 6–7 months contained non-animal-source foods. Among plant-based recipes, we found that the combination of grain and/or legume with fruits and/or vegetables was common for children aged 6–7 months. This type of meal is bulky with relatively low energy and nutrients, such as iron and zinc, and is high in anti-nutrients, such as phytate. From our ingredient analysis, we found that green leafy vegetables and dark yellow roots and tubers were commonly used in shared recipes for children aged 6–7 months. This result aligns with a previous study on recipes on “mom food blogs” showing that deep yellow vegetables and dark green vegetables were commonly used in vegetable recipes [14]. Even though green leafy vegetables are a rich source of iron, the bioavailability is low [42]. Moreover, an inappropriate combination of recipes containing non-animal-source foods could lead to low protein consumption. Rice has a low protein content with limited essential amino acids, lysine and threonine, whereas legumes have a low methionine content [42]. Therefore, rice should be mixed with legumes to ensure the source of essential amino acids. However, this plant-based combination seems to be high in phytate, interfering with iron absorption.

Our findings indicate that online members are concerned about food seasoning in home-cooked meals for children. Avoiding sugar, salt, and sauce was seen in shared recipes for children under one year old, while low-sodium cooking sauce or powder was more likely to be discussed in shared recipes for children aged one year old and over. Increasingly, evidence suggests that sugar and salt intake in childhood are associated with some adverse health behavior and outcomes, including the development of salty and sweet preferences, increasing the risk of high blood pressure and obesity [43,44,45,46]. Advice on complementary feeding by health experts suggests that sugar and salt should be avoided in complementary food [1,47].

Some of the parental practices embedded within peer-exchanged discourse were consistent with the complementary feeding guideline in the Thai’s Maternal and Child Health (MCH) Handbook [48]. We found that most food recipes contained animal-sourced foods with grain or legume and vegetable or fruit. These findings are consistent with the recommendation of the MCH handbook that parents should offer mixed meals of rice with meats and vegetables. The guideline recommends offering egg yolk to children aged six months old, and this food was commonly used in recipes for infants aged 6–7 months analyzed here. Moreover, the guideline recommended parents should avoid using food seasoning in complementary foods for children under one year old, which aligns to the online groups’ common practices. However, whereas guidelines recommend introducing whole egg at around seven months of age, whole egg was predominately used in shared recipes from ten months of age. Furthermore, although the recommendations suggest including some meat in every meal for children from six months old, we found that some recipes for young children were non-animal-source foods. These practices reflected parental concerns about food allergies. To properly address these concerns, it might be necessary to develop guidelines or instructions on introducing solid food to babies for allergy prevention.

Homemade foods offer an opportunity for parents not only to modify ingredients to provide adequate nutrients and avoid salt and sugar exposure, but also to promote taste development for children. Infants are born with an innate preference for sweet tastes and rejection of bitter or sour tastes [49]. Offering sweet-tasting complementary foods would enhance the preference of sweet flavor and diminish acceptance of less-sweet food such as vegetables. However, appropriate taste preferences may be promoted in the first two years of life [50]. Exposure to a greater variety of flavors or foods could promote the acceptance of novel foods [8]. The inborn bitter taste rejection could be modified by repeated exposure to a variety of vegetables. Our study found that the shared recipes had a vegetable variety score per meal of 2.0, which means that two types of vegetables were used per meal in general. The shared recipes by online peers in Thailand had the same average range of vegetables per meal as in recipes from cookbooks in the UK [22]. It should be noted that even though there was no limitation on the quality of food applied in the vegetable variety score calculation, some vegetables (e.g., spring onion, shallot, and lemongrass) used for food garnishing or considered as herb in Thai cuisine were not counted in the calculation. Although the previous study found that commercial meals had a greater vegetable variety per meal than home-cooked recipes [22], home-cooked foods were associated with less food allergy development [51]. The limiting factor of vegetable variety per meal could be improved by encouraging parents to increase the variety of vegetables in home-cooked foods.

Our study suggests that members use online peer-support communities as a platform for sharing and recommending food products used in food recipes for infants and young children. We found that processed meats and food seasoning were among the common topics around food products. This finding aligns with our unpublished previous survey study showing that parents who joined these sites were more likely to provide children with processed meats. There is growing evidence that processed meats consumption is associated with some adverse outcomes among preschool children, such as increased waist circumstance and cholesterol level [52,53]. Another study on online recipes has raised an issue that recipes on food blogs are excessive in saturated fat and sodium [54]. However, we found that the conversations around food products were focused solely on selecting the reduced sugar or sodium or additive-free products.

The present findings should be interpreted in light of a few limitations. First, the shared recipes in this study might not reflect the entire characteristics of online recipes for young children in the Thai context due to the single snapshot from five online communities. Second, the calculation of energy and nutrients was not conducted due to the lack of information regarding ingredient quantity and serving size. This limits the nutritional comparison between shared recipes and recipes from other sources. Another limitation is that this study only captured meals rather than a child’s full daily dietary consumption. These results do not reflect individual dietary diversity. Further, because this research relied on online data, parental beliefs and perceptions around complementary feeding foods might be different from those of the general population of mothers. A recent study examining sociodemographic predictors of parents’ use of the Internet for information on their child’s health and feeding found that nonworking mothers were more likely to seek child feeding information from the Internet [55]. However, there is little literature on the use of Internet or online groups for parental child feeding according to parents’ sociodemographic characteristics. Finally, the study would have been strengthened by more rigorous qualitative methods, such as member checking.

## 5. Conclusions

Online communities provide a new platform to encourage parents and caregivers to make home-cooked meals for children, which is seen as a healthier choice than commercial foods. Therefore, developing an understanding of parental perceptions of food choices would help guide healthcare professionals to better harness this existing online platform more effectively. Findings from this mixed-methods study suggested that user-generated content around shared recipes for infants and young children disseminated food choices that were seen as appropriate. Our finding enhances the understanding that parental food choices are embedded in the concept of not harming children. The introduction of first-time foods was driven by parental perceptions and concerns about food allergies. This practice resulted in delayed introduction of animal-source foods, including some common allergenic foods, which may contribute to low consumption in some essential nutrients for growth and development. There was also a concern about sugar, salt, and cooking sauce in children’s diets. The relaxation of the constraints on food seasoning when a child reached a year led to parents’ attention to exchange their opinion and recommendation on food products, including cooking sauce and processed meat products. Because of the potential learning environment, further study is required in addressing the impact of existing online communities on actual feeding practices.

## Figures and Tables

**Table 1:** Characteristics of shared recipes by children’s age groups.

Recipe Characteristics	Overall *n* = 379	Children’s Age Groups, Months					*p*
		6–7 *n* = 91	8–9 *n* = 74	10–11 *n* = 35	12–23 *n* = 148	≥24 *n* = 31	
Recipe type							
Animal source-containing, *n* (%)	344 (90.8)	61 (67.0)	71 (95.9)	33 (94.3)	148 (100.0)	31 (100.0)	<0.001 ^4^
Non-animal source-containing, *n* (%)	35 (9.2)	30 (33.0)	3 (4.1)	2 (5.7)	0 (0.0)	0 (0.0)	
Recipe combination ^1^							
Grain/Legume OR Vegetable/Fruit, *n* (%)	8 (2.1)	8 (8.8)	0 (0.0)	0 (0.0)	0 (0.0)	0 (0.0)	<0.001 ^4^
Flesh foods/Egg/Dairy, *n* (%)	10 (2.6)	0 (0.0)	0 (0.0)	0 (0.0)	8 (5.4)	2 (6.5)	
Grain/Legume + Vegetable/Fruit, *n* (%)	27 (7.1)	22 (24.2)	3 (4.1)	2 (5.7)	0 (0.0)	0 (0.0)	
Flesh foods/Egg/Dairy + Grain/Legume, *n* (%)	34 (9.0)	3 (3.3)	4 (5.4)	3 (8.6)	19 (12.8)	5 (16.1)	
Flesh foods/Egg/Dairy + Vegetable/ Fruit, *n* (%)	74 (19.5)	9 (9.9)	10 (13.5)	6 (17.1)	40 (27.0)	9 (29.0)	
Flesh foods/Egg/Dairy + Grain/Legume + Vegetable/Fruit, *n* (%)	226 (59.6)	49 (53.8)	57 (77.0)	24 (68.6)	81 (54.7)	15 (48.4)	
Vegetable variety score (VegVS) ^2^							
VegVS, mean ± *SD*	1.8 ± 1.5	1.5 ± 1.7	2.0 ± 1.5	2.2 ± 1.5	1.8 ± 1.4	1.4 ± 1.1	0.057 ^5^
VegVS, median (IQR ^3^)	2.0 (0.0–3.0)	1.0 (0.0–3.0)	2.0 (1.0–3.0)	2.0 (1.0–3.0)	2.0 (1.0–3.0)	1.0 (1.0–2.0)	

^1^ Combinations of recipes grouped by food types, not including oil, condiment, and breast milk; ^2^ VegVS: number of different vegetables within each recipe; ^3^ IQR= interquartile range; ^4^ Chi-square; ^5^ ANOVA.

## Data Availability

Data sharing not applicable.
